# Deep Brain Stimulation Lead Functional Repositioning After Spontaneous Pneumocephalus Resorption: A Clinical Case Presentation and Systematic Review

**DOI:** 10.7759/cureus.77506

**Published:** 2025-01-15

**Authors:** Yeimy Margarita Lebrón Sánchez, Viviana Torres, Angel Carreras, Alejandro A Jimenez Marrero, Ruben Dario Bleubar Ozoria, Lianca Rivera, Ambar Pérez-Fernández

**Affiliations:** 1 Parkinson's Disease and Movement Disorders Unit, Neurofunctional Group, Santo Domingo, DOM; 2 Institute of Human Anatomy, Autonomous University of Santo Domingo (UASD), Santo Domingo, DOM; 3 Medical School, Technological Institute of Santo Domingo (INTEC), Santo Domingo, DOM

**Keywords:** deep brain stimulation, electrode displacement, lead relocation, parkinson's disease, pneumocephalus

## Abstract

Deep brain stimulation (DBS) has become a critical intervention for managing advanced Parkinson's disease (PD), particularly for patients whose symptoms are no longer controlled by medication. This report details the case of a 61-year-old male with PD who experienced electrode displacement due to pneumocephalus following DBS surgery targeting the subthalamic nucleus (STN). Initial imaging revealed a significant subdural air volume causing electrode displacement. However, one month later, spontaneous pneumocephalus resorption led to the functional repositioning of the electrodes, restoring proper function and negating the need for reoperation. The accompanying systematic review analyzed 24 studies, involving 1,439 patients across 12 countries, to assess the occurrence and management in this specific scenario. Findings showed electrode displacement occurred in 75% of cases, but spontaneous repositioning happened only in 12.5%, typically with air volumes below 10 cm³. Larger volumes often required surgical intervention, though definitive thresholds for action remain unclear. The review highlights inconsistencies in managing this complication, emphasizing the need for clearer protocols to improve outcomes. This work underscores the rarity of spontaneous electrode realignment and the importance of careful evaluation of pneumocephalus volume and patient symptoms. It advocates for evidence-based management strategies to balance clinical intervention with the potential for natural resolution, aiming to enhance DBS efficacy and patient quality of life. Further research is necessary to establish standardized guidelines for addressing this complication.

## Introduction

Parkinson's disease (PD) is a neurodegenerative disorder that affects approximately 1-3% of the population over the age of 60, significantly reducing their quality of life [1,2]. As the disease advances, patients often experience debilitating motor and non-motor symptoms, which can severely impair daily functioning and independence. While pharmacological treatments, primarily dopaminergic therapies, are effective in managing symptoms during the early stages, their efficacy diminishes over time, leading to fluctuations and complications.

In this context, deep brain stimulation (DBS) has emerged as a promising intervention for patients with advanced PD who are no longer adequately managed with medication alone [1-3]. DBS involves the implantation of electrodes in specific brain regions, which deliver electrical impulses to modulate neural activity and alleviate motor symptoms. This technique has been shown to substantially improve motor function and quality of life for appropriately selected patients.

Despite its benefits, DBS is a complex procedure with risks and possible complications related to the device, the procedure, electrical stimulation, and current spread. These complications may include intracerebral hematomas, intracranial hemorrhages, ischemic stroke, seizures, dyskinesias, dysarthria, mood changes, Horner's syndrome, as well as issues with the hardware such as migration, erosion, or rupture of the electrodes. Notably, the development of pneumocephalus - characterized by the accumulation of air within the skull following craniotomy - can also occur [4-6].

Pneumocephalus can lead to neurological symptoms, and, in the context of DBS, it may displace implanted electrodes, compromising the treatment’s effectiveness. Managing a pneumocephalus-induced electrode displacement requires careful evaluation and sometimes reoperation to restore optimal function.

This article presents a clinical case involving a 61-year-old male patient with PD treated with DBS of the subthalamic nucleus (STN). Twelve hours after surgery for DBS placement, a follow-up computed tomography (CT) scan revealed the presence of pneumocephalus with the subsequent electrode displacement, leading to the decision for reoperation. One month later, a magnetic resonance imaging (MRI) scan with DBS protocols in preparation for the new surgery demonstrated complete resorption of the pneumocephalus and proper alignment of the electrodes with the previously planned coordinates. Following this, the device was activated, and the patient exhibited very good control of his PD symptoms.

In light of this case, we proposed this systematic review to provide valuable insights into managing DBS electrode displacement due to pneumocephalus. Specifically, it will address two critical questions that could influence decision-making in this scenario: 1) how often does the position of DBS electrodes in the STN spontaneously correct after the appearance and resorption of pneumocephalus? and 2) what are the guidelines for managing DBS electrode displacement after the occurrence of pneumocephalus? By exploring these questions, we aim to enhance clinical decision-making and optimize patient outcomes in DBS procedures.

This article was previously presented at the XLI Latin American Congress of Neurosurgery (CLAN) 2024 on October 22, 2024, titled Review of DBS Complications.

## Case presentation

We present the case of a 61-year-old male with an 11-year history of Parkinson's disease, classified as Hoehn and Yahr stage III. The patient exhibited severe global bradykinesia, handwriting impairment, freezing of gait, and rigidity, along with significant motor and non-motor fluctuations despite treatment with levodopa/carbidopa, pramipexole, amantadine, and clonazepam. The total Levodopa Equivalent Daily Dose (LEDD) was 1000 mg/day. Neurological examination revealed mild hypophonia, hypomimia, and moderate to severe bradykinesia with a slight resting tremor. Given the patient's severe motor fluctuations and satisfactory response to levodopa (61% improvement), we recommended bilateral DBS in the subthalamic nucleus to manage his symptoms.

**Figure 1:**
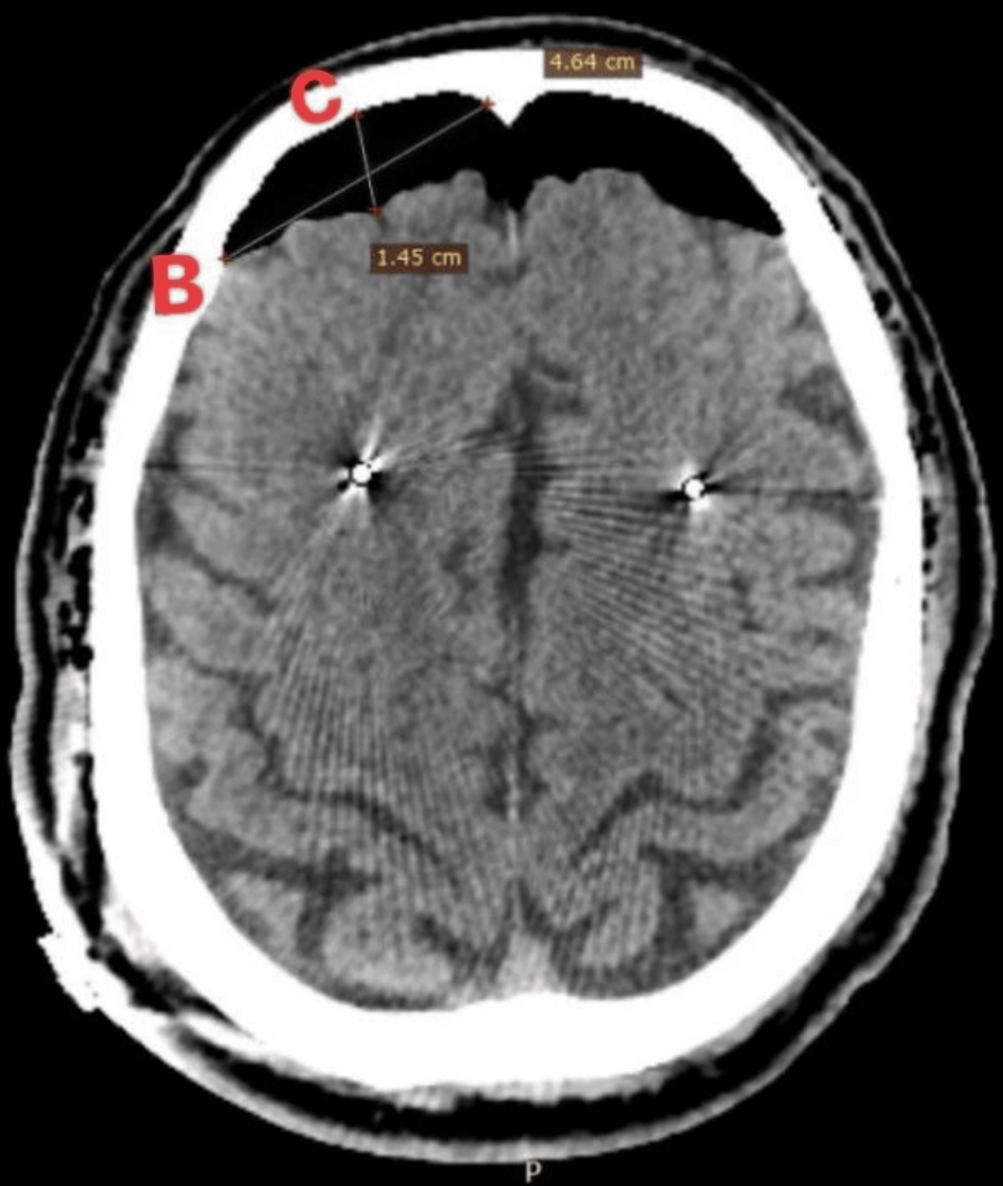
Early post-operative CT scan without contrast, showing subdural pneumocephalus of 25.11 cm³ A: the number of air pockets noted x section thickness, B: widest diameter in axial section, C: widest diameter perpendicular to B.

The DBS implantation surgery was performed while the patient was awake and guided by microelectrode recording (MER), adhering closely to the planned coordinates throughout the procedure. Following the surgery, a follow-up CT scan revealed significant pneumocephalus. Subdural air volume was 25.11 cm³ (Figure 1), estimated in brain CT according to the following formula: A x B x C / 2 [7]. The mean distance between initial and delayed DBS lead tip position was <2 mm. At the time, the patient's clinical condition remained stable and without neurological deficits.

Given these findings, the decision was made to reoperate. One month later, a new MRI performed in preparation for reintervention showed complete reabsorption of the pneumocephalus and realignment of the electrodes with the planned coordinates. Following this, stimulation was initiated and the patient’s parameters were adjusted to optimize therapeutic effects, resulting in better control of his symptoms. This improvement led to the decision to cancel the planned second surgery.

**Figure 2:**
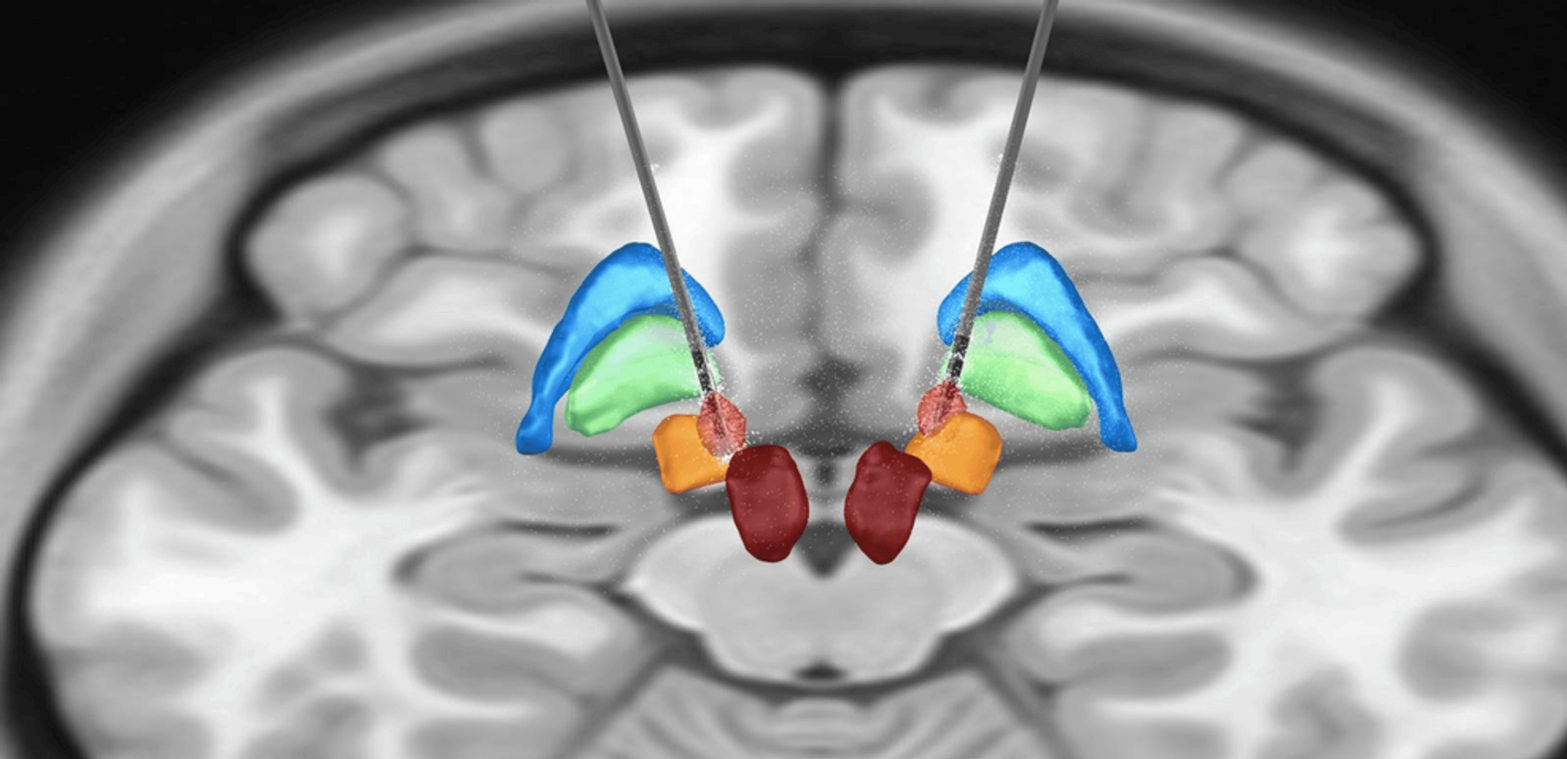
3D reconstruction model of final DBS-lead position and VTA DBS-lead: deep brain stimulation lead. VTA: volume of tissue activated (indicated in light red). Figure created by the authors using Lead-DBS (MATLAB) [9]

Lead-DBS [8,9] was used for 3D visualization of the final position of the electrodes and their relationship with the STN. Preoperative MRI and the latest CT scan were fused to establish the final electrode locations after air resorption. Additionally, a volume of tissue activated (VTA) model was constructed based on the stimulation parameters used, corroborating stimulus to the sensorimotor region of the STN, which correlates with the observed motor improvement (Figure 2).

Ethical considerations

Informed consent was obtained from the patient to present his results to the scientific community following confidentiality principles and measures to safeguard the patient's physical and emotional well-being. This process adhered to the guidelines outlined in the Declaration of Helsinki regarding medical research involving human subjects [10].

## Discussion

Literature search

This systematic review followed the Preferred Reporting Items for Systematic Reviews and Meta-Analyses (PRISMA) guidelines [11]. We aimed to evaluate the management of DBS electrode displacement due to pneumocephalus in patients with Parkinson's disease.

A comprehensive literature search was performed. The search terms included a combination of keywords (syntax search): ((electrode) OR (lead)) AND ((pneumocephalus) AND (DBS) AND (Parkinson)) in the PubMed, Google Scholar, Cochrane Library, and Global Index Medicus databases. Studies that did not meet the research objectives, theses, monographs, books, animal studies, and studies not published in English or Spanish were excluded. These searches were conducted without date restrictions.

Methodological quality assessment

The quality of the selected articles was evaluated using the Newcastle-Ottawa Scale (NOS), which has shown reliability and validity for structured and standardized assessment of the quality of cohort and case-control studies based on content, design, and usability [12]. This scale consists of eight items, divided into three dimensions (comparison, selection, study type), where each study can receive a maximum of nine points or "stars." High-quality studies can score 7-9 stars, moderate quality 4-6 stars, and low quality 0-3 stars.

Data extraction, management, and analysis

From the collected literature, a clear set of data items were stablished to answer the review questions. These items were recorded in a spreadsheet and evaluated by three independent reviewers, using easy coding rules and response options to ensure consistent responses among them and prevent potential sources of bias or systematic errors. Criteria for unifying responses in fields such as median pneumocephalus volume and total lead deflection for each study were discussed academically with the other authors to reach consensus, and frequent comparisons were made to limit errors in interpreting the data items.

For each article, the following were identified: year of publication, number of patients included, country, language, target/planning, pneumocephalus volume, total lead deflection, reintervention (yes/no), waiting time before reintervention, spontaneous repositioning (yes/no), severity of the patient's disease before and after surgery as assessed by the Unified Parkinson's Disease Rating Scale (UPDRS) [13], and patient symptoms before and after DBS.

A descriptive statistical analysis [14] was performed on the variables identified in the articles, presenting their absolute frequencies (number of cases) and relative frequencies (percentages), calculated using the JASP 0.18.1 software [15].

Systematic review results

Study Characteristics

**Figure 3:**
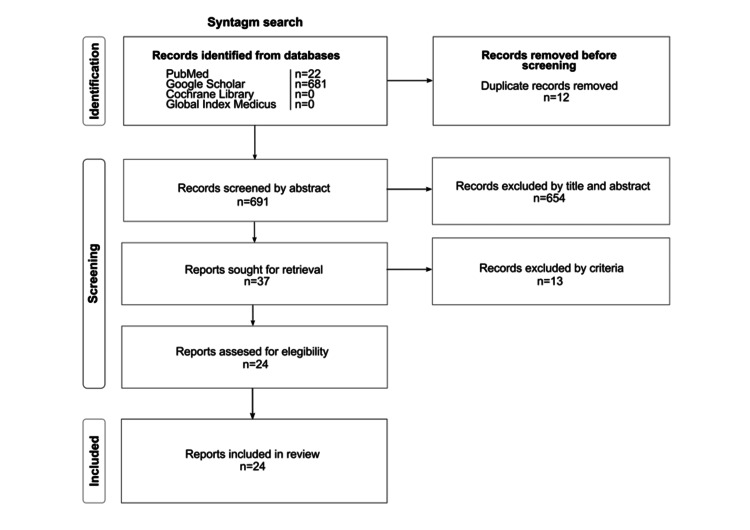
Search strategy according to the scheme proposed in the PRISMA 2020 guidelines. PRISMA: Preferred Reporting Items for Systematic Reviews and Meta-Analyses.

The systematic search procedure yielded 24 articles through the phases of identification, screening, and inclusion using syntax-based search (Figure 3).

A total of 24 articles were selected, comprising one case report and 23 cohort studies, all written in English, considering a total of 1439 patients who received DBS for the treatment of Parkinson's disease in 12 countries (Table 1). Of these, eight studies (33.3%) were conducted in Europe, 10 in North America (41.6%), and six (25.0%) in Asia.

**Table 1:** Publication count by country.

Country	n (%)
USA	10 (41.7%)
China	3 (12.5%)
Netherlands	2 (8.3%)
Austria	1 (4.1%)
Zurich	1 (4.1%)
Japan	1 (4.1%)
Spain	1 (4.1%)
Turkey	1 (4.1%)
Italy and USA	1 (4.1%)
Sweden	1 (4.1%)
South Korea	1 (4.1%)

Methodological Quality and Publication Frequency

All cohort studies demonstrated excellent methodological quality according to the NOS (7-9 stars); one study received 9 stars (4.3%), 17 studies (73.9%) received 8 stars, and five studies (21.7%) received 7 stars (Table 2). This scale is not defined for case reports.

**Table 2:** Articles included in review indicating the number of points or stars obtained in the parameters evaluated with the Newcastle-Ottawa Scale. *The article by Martinez-Nunez et al. (2024) is a case report, and the Newcastle-Ottawa Scale does not apply to single case studies that lack a comparative design.

ID	Year of publication	Authors	Selection (max=4)	Comparability (max=2)	Exposure (max=3)	Total (max=9)
1	2007	Miyagi et al. 2007 [16]	3	2	3	8
2	2008	Halpern et al. 2008 [17]	3	2	2	7
3	2009	Richardson et al. 2009 [18]	3	2	3	8
4	2010	Kim et al. 2010 [19]	3	1	3	7
5	2010	Nazzaro et al. 2010 [20]	3	2	3	8
6	2010	van den Munckhof et al. 2010 [21]	3	2	3	8
7	2011	Azmi et al. 2011 [22]	3	2	3	8
8	2014	Seijo et al. 2014 [5]	3	2	3	8
9	2015	Sharim et al. 2015 [23]	3	2	3	8
9	2018	Matias et al. 2018 [24]	3	2	3	8
10	2017	Bentley et al. 2017 [25]	3	1	3	7
11	2018	Matías et al. 2018 [24]	3	2	3	8
12	2020	Albano et. al. 2020 [26]	3	1	3	7
13	2020	Gong et al. 2020 [27]	3	2	3	8
14	2020	Niederer et al. 2020 [28]	3	2	3	8
15	2021	Göransson et al. 2021 [29]	3	2	3	8
16	2021	Krauss et al. 2021 [30]	3	2	3	8
17	2021	Piacentino et al. 2021 [31]	3	2	3	8
18	2021	Taskin et al. 2021 [32]	3	2	3	8
19	2021	van den Munckhof et al. 2021 [33]	3	2	3	8
20	2022	Hart et al. 2022 [34]	3	2	3	8
21	2023	Wu et al. 2023 [35]	3	2	3	8
22	2023	Yuan et al. 2023 [3]	3	2	3	8
23	2024	Chee et al. 2024 [36]	4	2	3	9
24	2024	Martinez-Nunez et al. 2024 [37]	*	*	*	*

Regarding the frequency of publication per year, 24 publications were found between 2007 and 2024. Of these, 11 (45.8%) appeared sporadically before 2020 (0.84 publications per year), but between 2020 and 2024, 54.2% of the publications were found, with a frequency of 2.1 publications per year.

The cohort studies on which this review is based demonstrated excellent methodological quality according to the NOS criteria. One study (4.3%) achieved the maximum score of 9 stars due to its high level of rigor in participant selection, group comparability, and outcome measurement [36]. Most studies (73.9%) received 8 stars because none included a selection of an unexposed cohort, a criterion that involves comparing outcomes with a group of DBS patients who did not develop pneumocephalus. Five studies (21.7%) received 7 stars, indicating good methodological quality, with minor comparability or patient follow-up deficiencies. The Newcastle-Ottawa Scale is not designed to evaluate case reports, as it only applies to studies lacking a comparative design, such as case studies, thus limiting its utility in such contexts.

Surgical Outcomes

Regarding the volume of the pneumocephalus, it was reported in 14 out of 19 studies (73.7%), of which 13 reported volumes ranging between 0.18 and 54.29 cm³. One study conducted in China presented unilateral pneumocephalus volume percent (uPVP), which refers to the percentage of the affected cerebral hemisphere's volume concerning the total volume (Table 3).

**Table 3:** Surgical outcomes: volume of pneumocephalus, electrode displacement and self-repositioning. NS: Not specified. S: Standard deviation. Min: Minimum. Max: Maximum. uPVP: unilateral pneumocephalus volume percent. *Yuan et al. reported the unilateral pneumocephalus volume percent (uPVP), which represents the percentage of the pneumocephalus volume in relation to the total volume of the affected cerebral hemisphere.

ID	Year	n	Authors	Volume in cm^3^*	Is there electrode displacement outside the target?	Did the electrode spontaneously reposition itself?	Total lead deflection (mm)
1	2007	18	Miyagi et al. 2007 [16]	NS	Yes	No	Mean±SD. 2.23±0.93 in Y axis and 0.39±0.68 in Z Axis.
2	2008	50	Halpern et al. 2008 [17]	NS	Yes	No	2 mm in Y axis.
3	2009	8	Richardson et al. 2009 [18]	NS	Yes	No	NS
4	2010	53	Kim et al. 2010 [19]	Mean±SD (Min-Max) 11.9±16.6 (0-76)	Yes	Yes	Mean±SD 0.6±0.5 in X axis, 1.1±0.9 in Y axis, and 1.0±0.8 in Z axis.
5	2010	61	Nazzaro et al. 2010 [20]	Mean±SD 0.98±1.42	No	No	NS
6	2010	14	van den Munckhof et al. 2010 [21]	Mean±SD 17±24	Yes	No	Mean±SD. 3.3±2.5
7	2011	32	Azmi et al. 2011 [22]	Mean±SD 13.57±12.44	Yes	No	0.88-1.33
8	2014	233	Seijo et al. 2014 [5]	Mean 20	Yes	No	NS
9	2015	85	Sharim et al. 2015 [23]	Mean±SD 21.49±13.70	No	No	NS
10	2017	73	Bentley et al. 2017 [25]	Mean±SD (Min-Max) 21.3±13.7 (0.1-65.9)	Yes	No	Mean±SD 2.0±1.2
11	2018	20	Matias et al. 2018 [24]	Median = 0.6, Min-Max: 0-32	No	No	NS
12	2020	2	Albano et. al. 2020 [26]	Patient 1: 54.29; Patient 2: 11.8	Yes	No	NS
13	2020	130	Gong et al. 2020 [27]	Mean±SD 10.1±12.6	Yes	Yes	Mean±SD 0.76±0.24
14	2020	33	Niederer et al. 2020 [28]	Mean±SD 8.8±5.6	Yes	No	Mean±SD 0.55±0.49 in X axis, 0.36±0.42 in Y axis, and 0.43±0.58 in Z axis.
15	2021	23	Göransson et al. 2021 [29]	Mean (Min-Max) of two groups. Left side: 0.19 (0–8.76) Right side: 0.18 (0-2.01)	Yes	No	Mean±SD 0.44±0.72 in X axis, 0.64±0.54 in Y axis, and 0.62±0.71 in Z axis.
16	2021	100	Krauss et al. 2021 [30]	Mean±SD 1.3±2.8	No	No	NS
17	2021	46	Piacentino et al. 2021 [31]	Mean of two groups. Supine position: 10.55, Semi-sitting position: 7.60	Yes	No	3.0 mm in X axis and 2.2 mm in Y axis.
18	2021	30	Taskin et al. 2021 [32]	Mean of two groups. Bone cement: 12.1, Stimloc®: 13.9	Yes	No	NS
19	2021	NS	van den Munckhof et al. 2021 [33]	NS	NS	NS	NS
20	2022	38	Hart et al. 2022 [34]	NS	Yes	No	0.22±0.4 with only 3 (4%) electrodes out with 2 mm from the intended target.
21	2022	255	Wu et al. 2022 [35]	Mean±SD of two groups. Elevated-head positioning group: 16.76±15.23, Supine positioning group: 3.25±8.78	NS	No	NS
22	2023	88	Yuan et al. 2023 [3]	uPVP* median of two groups. Cannula Puncture Group: 0.15% Cross-Incision: 0.50%	Yes	No	Mean±SD 0.29±0.44 in X axis, 0.62±0.39 in Y axis and not significant in Z axis.
23	2024	46	Chee et al. 2024 [36]	Mean±SD. 4.49±6.05	Yes, but less than 1mm.	No	NS
24	2024	1	Martinez-Nunez et al. 2024 [37]	NS	Yes, intentionally during the surgery.	Yes	1.4 medial and 1.4 posterior.

In this systematic review, the subthalamic nucleus was the most frequently reported target, as it is the most commonly operated site in these procedures (Table 4).

**Table 4:** Clinical outcomes and characteristics of electrode displacement and spontaneous repositioning in the reviewed studies. STN: subthalamic nucleus. GPi: globus pallidus internus. VIM: ventral intermediate nucleus.

Clinical results	n (%)
Target	
STN	16 (66.7%)
STN / GPi	2 (8.3%)
GPi / STN / VIM	1 (4.2%)
GPi / VIM	1 (4.2%)
VIM / STN	1 (4.2%)
GPi	1 (4.2%)
Not specified	2 (8.3%)
Electrode displacement	
Yes	18 (75.0%)
No	4 (16.7%)
Not specified	2 (8.3%)
Spontaneous repositioning of the electrode	
Yes	3 (12.5%)
No	20 (83.3%)
Not specified	1 (4.2%)
Time before re operation	
Provided	6 (25.0%)
Not specified	18 (75.0%)
Volume category	
Les than 10 cm³	7 (30.4%)
10-20 cm³	7 (30.4%)
More than 20 cm³	3 (13.0%)
Not specified	7 (30.4%)

Electrode displacement was often observed among patients undergoing this surgery. Spontaneous repositioning of the DBS electrode associated with pneumocephalus resorption was reported in three out of the 24 papers reviewed, with a total lead deflection <2mm in all three axles, the most reported volume of pneumocephalus ranging between 10 and 20 cm³.

Takeaways

This report highlights the complexities and potential complications associated with DBS for PD, particularly regarding pneumocephalus and electrode displacement.

In our case, initial post-operative imaging revealed a concerning pneumocephalus volume, prompting a decision for reoperation. However, a follow-up MRI a month later showed complete reabsorption of the pneumocephalus and realignment of the electrodes, suggesting that not all instances of electrode displacement require immediate surgical correction. Despite these insights, there remains a notable gap in the literature regarding standardized protocols for managing DBS electrode displacement due to pneumocephalus.

The systematic review revealed inconsistencies in reported practices and outcomes, indicating a need for more clarity on when surgical intervention is warranted. Based on the review, we highlight the following key findings:

Pneumocephalus and Electrode Displacement

Most studies suggest that smaller volumes of pneumocephalus (less than 10 cm³) are typically associated with spontaneous resorption and electrode repositioning. In contrast, larger volumes (greater than 20 cm³) tend to result in persistent electrode displacement, often requiring surgical intervention. This highlights the importance of controlling intracranial air volume postoperatively, as each additional cubic centimeter of intracranial air is associated with approximately 0.024 mm of DBS electrode translocation [28].

Challenges Posed by Pneumocephalus in DBS

Pneumocephalus significantly impacts the stability and effectiveness of DBS, with several studies demonstrating a direct correlation between larger pneumocephalus volumes and increased electrode displacement in the early postoperative period [19,22,32]. These findings underscore the importance of preventing the accumulation of intracranial air as a strategy to avoid electrode displacement and related complications.

Postoperative Monitoring and Electrode Localization

Ongoing monitoring of electrode migration in the weeks after surgery is crucial. Van den Munckhof et al. [33] recommend regular postoperative assessments with extended follow-up, particularly for patients with large volumes of subdural air. Additionally, studies such as that by Kim et al. suggest performing brain CT scans at least one month after surgery to more accurately assess electrode localization. While immediate postoperative imaging is crucial for confirming initial electrode placement, recent studies suggest that delayed follow-up imaging may offer a more precise assessment of final electrode position, especially in cases of significant cortical displacement [26].

Discrepancies in the Relationship Between Pneumocephalus and Electrode Displacement

Despite the general association between pneumocephalus and electrode displacement, some recent research has questioned the clinical significance of this relationship. In a study by Chee et al. [36], pneumocephalus volumes up to 22.0 cm³ did not result in significant brain displacement compared to patients without pneumocephalus. The mean magnitude of displacement was less than 1.0 mm, regardless of intracranial air volume. These findings suggest that while pneumocephalus is often associated with electrode displacement, its clinical impact may not be significant in all cases, and other factors may contribute to electrode migration.

Clinical and Temporal Considerations

A practical scale proposed by Gong et al. [27] to assess DBS surgery outcomes reported that in one out of 130 patients (0.8%), electrodes spontaneously realigned with their intended target one week after pneumocephalus caused a posterior bending of the lead. Martinez-Nunez (2024) [37] also described a case in which pneumocephalus was detected during surgery, and a cannula was left in place to guide electrode repositioning. Although the frequency of publications on this issue has increased from 0.8 articles per year between 2007 and 2018 to an average of 2.5 articles between 2020 and 2023 [1,34], such cases remain relatively rare.

Reoperation Decision-Making

The decision to reoperate is typically driven by the lack of clinical improvement or the progression of symptoms attributed to pneumocephalus [26]. This symptom-based approach highlights the importance of close monitoring in the immediate postoperative period, with reoperation indicated if neurological symptoms worsen or if complications arise. On the other hand, electrode migration can be evaluated over up to one year [32]. However, there is no consensus on the optimal time for reassessment, with studies suggesting time frames ranging from a few days to one year for potential reoperation. This approach disparity underscores the need for clearer, more standardized guidelines for clinical decision-making in electrode displacement cases, considering both clinical progression and electrode stability.

Proposed Management Algorithm or Decision-Making Protocol

Given the lack of standardized protocols, we propose a management algorithm to guide decision-making in DBS electrode displacement caused by pneumocephalus.

**Figure 4:**
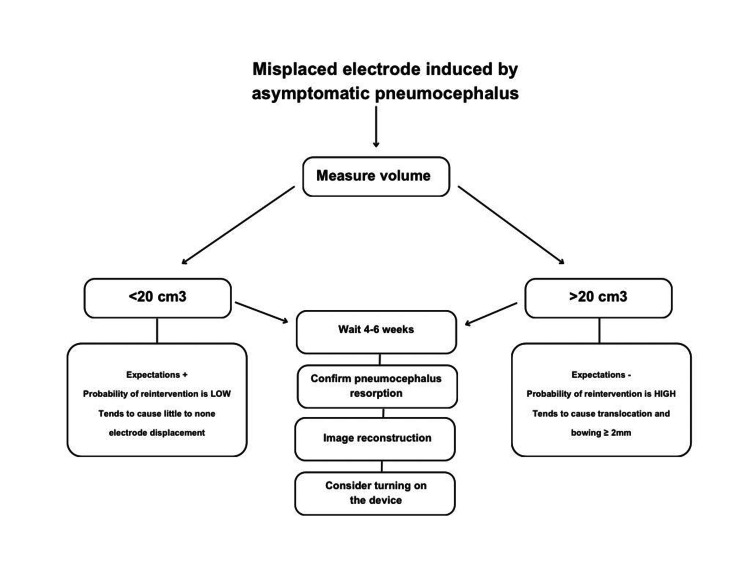
Decision-making protocol for electrode displacement management following deep brain stimulation surgery.

The protocol outlined in Figure 4 suggests a clinical approach for managing electrode displacement following DBS surgery, particularly due to pneumocephalus.

## Conclusions

Spontaneous DBS lead repositioning after pneumocephalus resorption is rare and depends on factors like intracranial air (ICA) volume. This review highlights a lack of evidence to guide decision-making in these cases, particularly in Latin America. Clinicians must balance clinical signs with imaging findings, as not all electrode displacements require surgery. Further research is needed to develop clear, region-specific protocols to guide management and follow-up, tailored to individual patient needs.

## References

[1] Bucur M, Papagno C (2023). Deep brain stimulation in Parkinson disease: a meta-analysis of the long-term neuropsychological outcomes. Neuropsychol Rev.

[2] Faggiani E, Benazzouz A (2017). Deep brain stimulation of the subthalamic nucleus in Parkinson's disease: from history to the interaction with the monoaminergic systems. Prog Neurobiol.

[3] Yuan T, Chen Y, Zhu G, Zhang J (2023). The related factors and effect of electrode displacement on motor outcome of subthalamic nuclei deep brain stimulation in Parkinson’s disease. J Clin Med.

[4] Fenoy AJ, Simpson RK Jr (2014). Risks of common complications in deep brain stimulation surgery: management and avoidance. J Neurosurg.

[5] Seijo F, Alvarez de Eulate Beramendi S, Santamarta Liébana E, Lozano Aragoneses B, Saiz Ayala A, Fernández de León R, Alvarez Vega MA (2014). Surgical adverse events of deep brain stimulation in the subthalamic nucleus of patients with Parkinson's disease. The learning curve and the pitfalls. Acta Neurochir (Wien).

[6] Vanamoorthy P, Tomar A, Prabhakar H, Rath GP (2018). Rare complications during deep brain stimulation surgery for Parkinson’s disease. J Neuroanaesth Crit Care.

[7] Broderick JP, Brott TG, Grotta JC (1994). Intracerebral hemorrhage volume measurement. Stroke.

[8] Treu S, Strange B, Oxenford S, Neumann WJ, Kühn A, Li N, Horn A (2020). Deep brain stimulation: imaging on a group level. Neuroimage.

[9] Horn A, Kühn AA (2015). Lead-DBS: a toolbox for deep brain stimulation electrode localizations and visualizations. Neuroimage.

[10] Asociación Médica Mundial. Declaración de Helsinki (2024). Declaración de Helsinki: Principios éticos para las investigaciones médicas en seres humanos. Internet.

[11] Page MJ, McKenzie JE, Bossuyt PM (2021). The PRISMA 2020 statement: an updated guideline for reporting systematic reviews. BMJ.

[12] (2024). The Newcastle-Ottawa Scale (NOS) for Assessing the Quality of Non-Randomized Studies in Meta-Analysis. https://www.ohri.ca/programs/clinical_epidemiology/oxford.asp.

[13] (2003). The Unified Parkinson's Disease Rating Scale (UPDRS): status and recommendations. Mov Disord.

[14] Daniel W, Cross CL (2013). Biostatistics: A Foundation for Analysis in the Health Sciences. https://www.wiley.com/en-us/Biostatistics%3A+A+Foundation+for+Analysis+in+the+Health+Sciences%2C+11th+Edition-p-9781119496571#description-section.

[15] (2024). JASP Team. JASP Team [Internet.

[16] Miyagi Y, Shima F, Sasaki T (2007). Brain shift: an error factor during implantation of deep brain stimulation electrodes. J Neurosurg.

[17] Halpern CH, Danish SF, Baltuch GH, Jaggi JL (2008). Brain shift during deep brain stimulation surgery for Parkinson's disease. Stereotact Funct Neurosurg.

[18] Richardson RM, Ostrem JL, Starr PA (2009). Surgical repositioning of misplaced subthalamic electrodes in Parkinson's disease: location of effective and ineffective leads. Stereotact Funct Neurosurg.

[19] Kim YH, Kim HJ, Kim C, Kim DG, Jeon BS, Paek SH (2010). Comparison of electrode location between immediate postoperative day and 6 months after bilateral subthalamic nucleus deep brain stimulation. Acta Neurochir (Wien).

[20] Nazzaro JM, Lyons KE, Honea RA (2010). Head positioning and risk of pneumocephalus, air embolism, and hemorrhage during subthalamic deep brain stimulation surgery. Acta Neurochir (Wien).

[21] van den Munckhof P, Contarino MF, Bour LJ, Speelman JD, de Bie RM, Schuurman PR (2010). Postoperative curving and upward displacement of deep brain stimulation electrodes caused by brain shift. Neurosurgery.

[22] Azmi H, Machado A, Deogaonkar M, Rezai A (2011). Intracranial air correlates with preoperative cerebral atrophy and stereotactic error during bilateral STN DBS surgery for Parkinson's disease. Stereotact Funct Neurosurg.

[23] Sharim J, Pezeshkian P, DeSalles A, Pouratian N (2015). Effect of cranial window diameter during deep brain stimulation surgery on volume of pneumocephalus. Neuromodulation.

[24] Matias CM, Frizon LA, Asfahan F, Uribe JD, Machado AG (2018). Brain shift and pneumocephalus assessment during frame-based deep brain stimulation implantation with intraoperative magnetic resonance imaging. Oper Neurosurg (Hagerstown).

[25] Bentley JN, Guan Z, Cummings KS, Chou KL, Patil PG (2017). Influence of intracranial air on electrode position and clinical outcomes following deep brain stimulation for Parkinson’s disease. Stereotact Funct Neurosurg.

[26] Albano L, Rohatgi P, Kashanian A, Bari A, Pouratian N (2020). Symptomatic pneumocephalus after deep brain stimulation surgery: report of 2 cases. Stereotact Funct Neurosurg.

[27] Gong S, Tao Y, Jin H (2020). Assessment of deep brain stimulation implantation surgery: a practical scale. World Neurosurg.

[28] Niederer J, Patriat R, Rosenberg O (2020). Factors influencing electrode position and bending of the proximal lead in deep brain stimulation for movement disorders. Stereotact Funct Neurosurg.

[29] Göransson N, Johansson JD, Wårdell K, Zsigmond P (2021). Postoperative lead movement after deep brain stimulation surgery and the change of stimulation volume. Stereotact Funct Neurosurg.

[30] Krauss P, Van Niftrik CH, Muscas G, Scheffler P, Oertel MF, Stieglitz LH (2021). How to avoid pneumocephalus in deep brain stimulation surgery? Analysis of potential risk factors in a series of 100 consecutive patients. Acta Neurochir (Wien).

[31] Piacentino M, Beggio G, Rustemi O, Zambon G, Pilleri M, Raneri F (2021). Pneumocephalus in subthalamic deep brain stimulation for Parkinson's disease: a comparison of two different surgical techniques considering factors conditioning brain shift and target precision. Acta Neurochir (Wien).

[32] Taskin O, Kocabicak E, Ozturk S, Yildiz O, Temel Y (2022). Electrode fixation with bone cement or stimloc® in deep brain stimulation surgery: a comparative study. Turk Neurosurg.

[33] van den Munckhof P, Bot M, Schuurman PR (2021). Targeting of the subthalamic nucleus in patients with Parkinson’s disease undergoing deep brain stimulation surgery. Neurol Ther.

[34] Hart MG, Posa M, Buttery PC, Morris RC (2022). Increased variance in second electrode accuracy during deep brain stimulation and its relationship to pneumocephalus, brain shift, and clinical outcomes: a retrospective cohort study. Brain Spine.

[35] Wu B, Xu J, Zhang C (2023). The effect of surgical positioning on pneumocephalus in subthalamic nucleus deep brain stimulation surgery for Parkinson disease. Neuromodulation.

[36] Chee K, Hirt L, Mendlen M (2024). Brain shift during staged deep brain stimulation for movement disorders. Stereotact Funct Neurosurg.

[37] Martinez-Nunez AE, Wong JK, Hilliard JD, Foote KD, Okun MS (2024). Preventing shift from pneumocephalus during deep brain stimulation surgery: don’t give up the ‘fork in the brain’. Tremor Other Hyperkinet Mov (N Y).

